# Highly expressed lncRNA H19 in endometriosis promotes aerobic glycolysis and histone lactylation

**DOI:** 10.1530/REP-24-0018

**Published:** 2024-07-02

**Authors:** Xiaoyang Wen, Jingyang Zhang, Zihan Xu, Muzi Li, Xiaotong Dong, Yanbo Du, Zhen Xu, Lei Yan

**Affiliations:** 1 Institute of Women, Children and Reproductive Health, Shandong University, Jinan, Shandong, China; 2 State Key Laboratory of Reproductive Medicine and Offspring Health, Shandong University, Jinan, Shandong, China; 3 Medical Integration and Practice Center, Shandong University, Jinan, Shandong, People’s Republic of China; 4 Shandong Key Laboratory of Reproductive Medicine, Jinan, Shandong, People’s Republic of China; 5 Reproductive Hospital Affiliated to Shandong University, Jinan, Shandong, People’s Republic of China; 6 Center for Medical Genetics and Prenatal Diagnosis, Shandong Provincial Maternal and Child Health Care Hospital, Jinan, Shandong, China; 7 Shandong Medicine and Health Key Laboratory of Birth Defect Prevention and Genetic Medicine, Jinan, Shandong, China; 8 Key Laboratory of Birth Regulation and Control Technology of National Health Commission of China, Jinan, Shandong, China

## Abstract

**In brief:**

Abnormal glucose metabolism may be involved in the pathogenesis of endometriosis. The present study identifies that highly expressed H19 leads to increased aerobic glycolysis and histone lactylation levels in endometriosis.

**Abstract:**

Previous studies from our group and others have shown increased IncRNA H19 expression in both the eutopic endometrium and the ectopic endometriosis tissue during endometriosis. In this study, we use immunofluorescence, immunohistochemistry, and protein quantification to determine that levels of aerobic glycolysis and histone lactylation are increased in endometriosis tissues. In human endometrial stromal cells, we found that high H19 expression resulted in abnormal glucose metabolism by examining the levels of glucose, lactate, and ATP and measuring protein levels of enzymes that participate in glycolysis. At the same time, immunofluorescence and western blotting demonstrated increased histone lactylation in H19 overexpressing cells. Altering aerobic glycolysis and histone lactylation levels through the addition of sodium lactate and 2-deoxy-d-glucose demonstrated that increased aerobic glycolysis and histone lactylation levels resulted in enhanced cell proliferation and cell migration, contributing to endometriosis. To validate these findings *in vivo*, we constructed an endometriosis mouse model, demonstrating similar changes in endometriosis tissues *in vivo*. Both aerobic glycolysis and histone lactylation levels were elevated in endometriotic lesions. Taken together, these data demonstrate elevated expression levels of H19 in endometriosis patients promote abnormal glucose metabolism and elevated histone lactylation levels *in vivo*, enhancing cell proliferation and migration and promoting the progression of endometriosis. Our study provides a functional link between H19 expression and histone lactylation and glucose metabolism in endometriosis, providing new insights into disease mechanisms that could result in novel therapeutic approaches.

## Introduction

Endometriosis, one of the most prevalent benign gynecologic diseases, is characterized by the presence of endometrial tissue, including glands and stroma, outside the uterus (Giudice & Kao 2004). Numerous theories and hypotheses have attempted to explain the etiology of endometriosis, including retrograde menstruation, which is currently favored by the literature (Vercellini *et al.* 2014). Therefore, understanding the physiological changes that occur in both the eutopic endometrium and the ectopic endometriosis tissue is critical for a more complete understanding of the disease. Our preliminary results (Xu *et al.* 2019) and the results of others (Liu *et al.* 2020) have shown that lncRNA H19 expression is elevated in both eutopic endometrium and ectopic endometriosis tissue.

Despite endometriosis being a benign condition, ectopic endometrial cells exhibit many characteristics thought to promote malignancy in cancer cells. For instance, the Warburg effect, a term describing a metabolic shift in cancer cells that makes them increasingly dependent on aerobic glycolytic pathways for ATP production (Handy *et al.* 2011), results in the production of metabolites that can enhance cell proliferation (Jaworska *et al.* 2023). This effect has been shown to promote endometriosis (Qi *et al.* 2014). Metabolic differences consistent with the Warburg effect have also been observed in endometriosis (Young *et al.* 2014). Hence, increased aerobic glycolysis might also be one pathogenic mechanism promoting endometriosis. Many enzymes participating in glycolysis, including fructose-6-phosphate-2-kinase/fructose-2,6-bisphosphatase 3 (PFKFB3), are highly expressed in endometriosis cells and contribute to its progression (Wang *et al.* 2021*b*). lncRNA H19, a member of a highly conserved imprinted gene cluster, has been shown to promote glycolysis in cancer cells (Gabory *et al.* 2010). This led us to examine if the high expression of H19 in endometriosis could alter the level of aerobic glycolysis.

Lactate production during glycolysis in cancer cells can affect cell proliferation, invasion, migration, and immune responses (Hirschhaeuser *et al.* 2011). In endometriosis, there is also an elevation in aerobic glycolysis and proliferation (Sun *et al.* 2022). Histone lactylation, a novel epigenetic modification recently described to promote gene transcription (Zhang *et al.* 2019), has been shown to promote tumorigenesis (He *et al.* 2023). However, no studies have examined histone lactylation for functional roles in modulating proliferation or migration in endometriosis. Therefore, we examined cellular metabolism, downstream changes in histone lactylation, and how these factors regulate disease expression in the context of altering H19 expression levels. This approach yielded a special function of H19, highlighting its potential as a therapeutic target for endometriosis.

## Materials and methods

### Patients and patient sample collection

Forty participants were enrolled at the Reproductive Hospital Affiliated to Shandong University from July 2021 to November 2022. Sample collection was approved by the Institutional Review Board (IRB) of the Reproductive Hospital Affiliated to Shandong University, and informed consent was obtained from all participants. Twenty patients in the disease group had been diagnosed with endometriosis during laparoscopic surgery. In comparison, 20 control samples from were obtained from patients of reproductive age treated for oviductal obstruction. None of the patients had a history of immune disorders, acute inflammatory disease, or estrogen-dependent diseases, and had not used hormonal drugs for at least 3 months prior to tissue collection. Samples were stored in liquid nitrogen and subsequently manipulated. Basic clinicopathological information is provided in Supplementary Table 1 (see section on supplementary materials given at the end of this article).

### Cell culture and cell transfection

Human endometrial stromal cells (HESCs ATCCCRL4003™) were a kind gift from Junhao Yan located at Shandong University. HESCs are immortalized endometrial stromal cells derived from uterine leiomyomas in adult women. The HESC culture conditions medium was DMEM/F12 (21041025, Gibco, USA) supplemented with 10% fetal bovine serum (FBS, 10091148, Gibco) and 1% penicillin–streptomycin. Cells were maintained at 37°C in 95% humidity, with 5% CO_2_ in a sterile tissue culture incubator.

Cells were processed when the cell density reached 75–85%. Transient H19 knockdown was performed by transfecting 10 nmol/L siRNA targeting human H19 (Life Technologies, Carlsbad, CA, USA) or non-specific control siRNA (Life Technologies) into HESCs for 48 h using Lipofectamine 3000 (L3000001, Invitrogen, USA) according to the manufacturer's instructions. To overexpress H19, HESCs were transfected with 3 μg H19 plasmid or 3 μg empty plasmid serving as control (GenePharma, Shanghai, China) for 48 h using Lipofectamine 3000, according to the manufacturer's instructions.

### Measurements of glucose consumption and lactate production

After HESC transfection, the cell medium was changed, and the medium was collected after 24 h for measurement. Each well contained a consistent number of cells. Glucose and lactate levels in the cell medium were measured using the Glucose Assay Kit (A154-1-1, Jiancheng Bio-technique Institute, China) or the Lactate Assay Kit (A019-2-1, Jiancheng Bio-technique Institute) following the reagent manufacturer's instructions.

### Measuring ATP content

The HESCs were collected 24 h after treatment according to the assay instructions. Intracellular ATP was then collected on ice using an Enhanced ATP Assay Kit (S0027, Beyotime, Shanghai, China). A dual-luciferase reporter assay system (Promega) was used to quantify normalized fluorescent signals according to the manufacturer’s protocols.

### Quantitative real-time PCR

RNA was obtained using TRIzol reagent (15596018, Ambion, USA) and reverse transcribed into cDNA. Quantitative real-time PCR was performed using the TaKaRa PrimeScript™ RT reagent Kit with gDNA Eraser (RR047Q, Takara, China). The sequences of primers are listed in Supplementary Table 2. The expression levels were normalized to those of the internal control β-actin and are presented as mean ± s.e.m. of at least three independent experiments.

### Protein extraction and western blotting

The HESCs were harvested and lysed on ice using radioimmunoprecipitation assay buffer (RIPA, Beyotime) mixed with the Protease/Phosphatase Inhibitor Cocktail (100×; #5872, Cell Signaling Technology, USA). The proteins were separated by a 4–20% Precast Protein Gel 15 Wells (SLE009, Smart Lifesciences, China), followed by transfer to a polyvinylidene difluoride membrane (IPVH00010, Merck, USA). After blocking with QuickBlock™ Blocking Buffer for Western Blot (P0252, Beyotime), the membrane was incubated with the primary antibody against the target protein on a shaking table at 4°C overnight. Then, membranes were washed and incubated with the appropriate secondary antibodies for 1 h at 37°C. Protein bands were detected by Clarity Western ECL Substrate (1705061, Bio-Rad, USA) and quantified using Quantity One software (Bio-Rad). The antibodies used are as follows: anti-tubulin (1:10,000, 66031-1-Ig, Proteintech, USA); anti-PKM2 (1:1000, 4053, Cell Signaling Technology); anti-LDHA (1:1000, 3582, Cell Signaling Technology); anti-ALDOA (1:1000, 11217-1-AP Proteintech); anti-L-lactyl lysine (pan kla) (1:1000, PTM-1401RM; PTM BIO); anti-L-lactyl-histone H3 (Lys18) (1:1000, PTM-1406RM; PTM BIO); anti-L-lactyl-histone H3 (Lys14) (1:1000, PTM-1414RM; PTM BIO); anti-H3 (1:5000, 68345-1-Ig, Proteintech); anti-L-lactyl-histone H3 (Lys9) (1:1000, PTM-1419RM; PTM BIO); horseradish enzyme-conjugated goat anti-rabbit IgG (H+L) (1:5000, ZB-2301, ZSGB-BIO, China); and horseradish enzyme-conjugated goat anti-mouse IgG (H+L) (1:5000, ZB-2305, ZSGB-BIO).

### Immunofluorescence staining of cell cultures and tissue and immunocytochemistry

Human normal endometrial tissue, eutopic and ectopic endometrial tissues in paraffin-embedded sections were deparaffinized, rehydrated, fixed, and blocked with QuickBlock Blocking Buffer (P0260, Beyotime). They were then incubated with the following primary antibodies: anti-L-lactyl lysine (pan kla) (1:100, PTM-1401RM; PTM BIO); anti-L-lactyl-histone H3 (Lys18) (1:100, PTM-1406RM; PTM BIO); anti-CD10 (1:250,14-0106-82, Invitrogen); and anti-H3 (1:1000, 68345-1-Ig, Proteintech) at 4°C overnight. Samples were washed three times with PBS, and then secondary antibodies (4412 and 8889, Cell Signaling Technology) diluted at 1:800 in Immunofluorescence Staining Secondary Antibody Dilution Buffer (P0108; Beyotime) were applied in a wet box for 1 h at room temperature in the dark. The tissues were mounted using the Anti-Fade Fluorescence Mounting Medium (ab104135, Abcam, USA) and stored at 4°C in the dark until analyzed. The fluorescence was visualized using a Leica inverted fluorescence microscope (Leica).

For immunocytochemistry, transfected treated cells were planted in 12-well plates containing Cover Glasses (801010, NEST, China). After fixing with 4% paraformaldehyde and incubating with PBS containing 0.5% Triton X-100, the process for endogenous peroxidase removal, and subsequent steps were the same as immunofluorescence.

### Immunohistochemical staining and hematoxylin–eosin

Tissues were embedded in paraffin, sectioned, and deparaffinized. Antigen repair was performed in a 100°C water bath using EDTA (PR30002, Proteintech). Permeabilization and blocking are done with The Rabbit Two-step Detection Kit (PV-9001, ZSGB-BIO), following the manufacturer's instructions. The slides were then incubated with primary antibodies overnight at 4°C. The antibodies used are as follows: anti-PKM2 (1:800, 4053, Cell Signaling Technology); anti-LDHA (1:500, 3582, Cell Signaling Technology); anti-ALDOA (1:200, 11217-1-AP, Proteintech); anti-CD10 (1:250, 14-0106-82, Invitrogen); anti-L-lactyl-histone H3 (Lys18) (1:100, PTM-1406RM; PTM BIO); and anti-L-lactyl lysine (pan kla) (1:100, PTM-1401RM; PTM BIO). The next day, secondary antibody (PV-9001, ZSGB-BIO) incubation and chromogenic detection were performed. The DAB Chromogenic Kit (ZLI-9018, ZSGB-BIO) was used according to the instruction manuals. The sections were then counterstained with hematoxylin, dehydrated, mounted, and examined.

Tissue sections for HE staining were deparaffinized and rehydrated. They were treated with hematoxylin for 50 s, ethanol containing 1% hydrochloric for 30 s, and then immersed in eosin for 2 min. Dehydration and subsequent steps were similar to immunohistochemistry. The tissue was photographed using an optical microscope.

### Cell proliferation assays

The cell proliferation was assessed at 0, 24, 48, and 72 h using Cell Counting Kit-8 (CCK-8) assay (C0039, Beyotime). Briefly, cells were plated into 96-well plates at a density of 5000 cells per well. Then, 10 µL CCK8 solution were added to each well, and cells were incubated for another 2 h at 37°C. The optical density (OD) at 450 nm was measured. Each group contained five replicates and all experiments were performed in triplicate.

### Transwell assays

Transwell assays were performed using 24-well plates with 8 µm pore size inserts (Corning Life Sciences, Corning, USA) according to the manufacturer’s protocols. In the migration assay, the HESCs (10^5^ cells/well) were seeded into the upper chamber in 200 µL serum-free, phenol red-free DMEM/F12 and allowed to migrate for 24 h to the lower chamber, which contained phenol red-free DMEM/F12 with 10% FBS. Transwell filters were fixed with 4% paraformaldehyde for 30 min, stained with 0.1% crystal violet for 15 min, and fixed on a glass slide. Cells that did not migrate were wiped off with cotton swabs. The cells that migrated to the lower chamber were imaged and counted.

### Modeling endometriosis in mice

Six- to eight-week-old female BALB/c mice were purchased from Vital River Co. Ltd, and all mice were housed in a specific-pathogen-free environment (humidity, 50 ± 5%; temperature, 20–22°C). Animals were randomly divided into two groups: donor and recipient. Donor mice were injected intramuscularly with 300 μg/kg estradiol benzoate (Sigma, USA) on days 1 and 4 to stimulate the growth of the endometrium. On day 7, the uteruses of donor mice were removed and minced, and then the tissue fragments were injected into recipient mice intraperitoneally. All recipient mice were sacrificed 14 days after implantation, and the endometriosis lesions were collected. Control mice were injected intraperitoneally with PBS in place of tissue fragments.

### Statistical analysis

Data are presented as mean ± s.e.m. Statistical analyses were performed using an unpaired two-tailed Student’s *t*-test and one-way ANOVA with GraphPad Prism 9. A statistical difference was observed when *P* < 0.05.

## Results

### Aerobic glycolysis is elevated in endometriotic lesions

**Figure 1:**
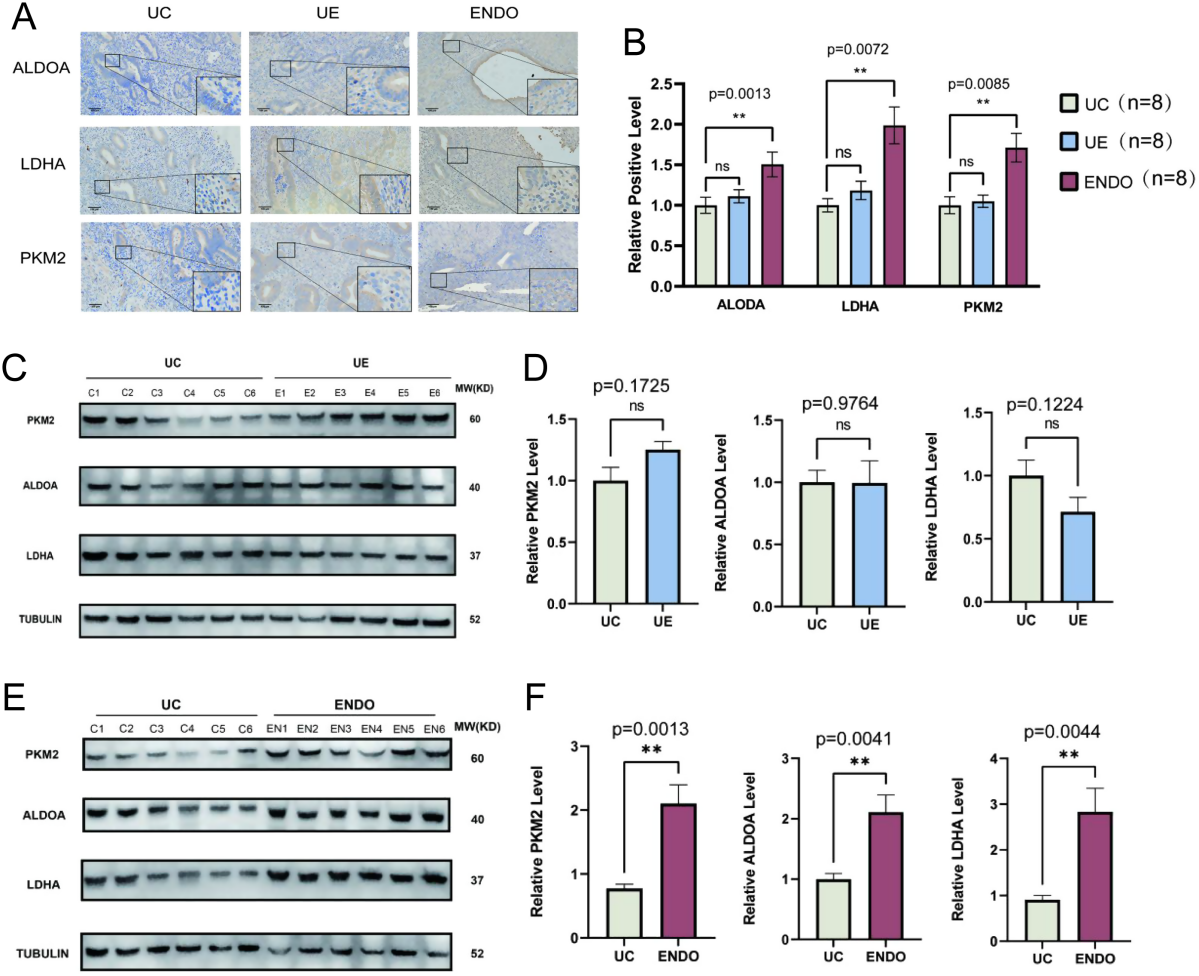
Aerobic glycolysis-related enzymes were expressed at elevated levels in ectopic endometriosis. (A) Immunohistochemical results showed increased expression of LDHA, PKM2, and ALDOA in ENDO compared to UC, which were mainly localized in the cytoplasm. Scale bar, 100 μm. (B) Quantitative analysis of (A). (C) Aerobic glycolysis-related enzymes levels were detected in UC and UE by western blot. There was no significant difference between UE compared to the UC. (D) Quantitative analysis of (C). (E) Aerobic glycolysis-related enzymes levels were detected in UC and ENDO by western blot. The expression of aerobic glycolysis-related enzymes was elevated in the ENDO group compared with the UC group. (F) Quantitative analysis of (E). The data are expressed as mean ± s.e.m. *P < 0.05, **P < 0.01, ***P < 0.001, and ****P < 0.0001; ns, no statistical difference.

To identify potential metabolic changes in eutopic endometrium and ectopic endometriosis tissue, the expression of glycolysis-related proteins was assayed using immunohistochemistry and western blotting. Immunohistochemical analysis of normal endometrium (UC), eutopic endometrium (UE), and ectopic tissue (ENDO) showed that lactate dehydrogenase A (LDHA), Aldolase (ALDOA), and pyruvate kinase M2 (PKM2) proteins were expressed by both endometrial glandular cells and stromal cells and were mainly localized in the cytoplasm. The expression of the LDHA, ALDOA, and PKM2 proteins was significantly higher in ectopic tissue (Fig. 1A and B). Meanwhile, western blotting confirmed that the expression of LDHA, ALDOA, and PKM2 were elevated at the protein level in ectopic tissue compared to normal endometrium (Fig. 1E and F). However, there were no significant differences in the expression levels of glycolysis-related proteins in UE compared to UC detected by immunohistochemistry (Fig. 1A, B, C, andD). These observations are consistent with previous findings that the levels of proteins participating in aerobic glycolysis are increased in the ectopic tissue formed in endometriosis.

### Histone lactylation levels are increased in endometriosis

**Figure 2:**
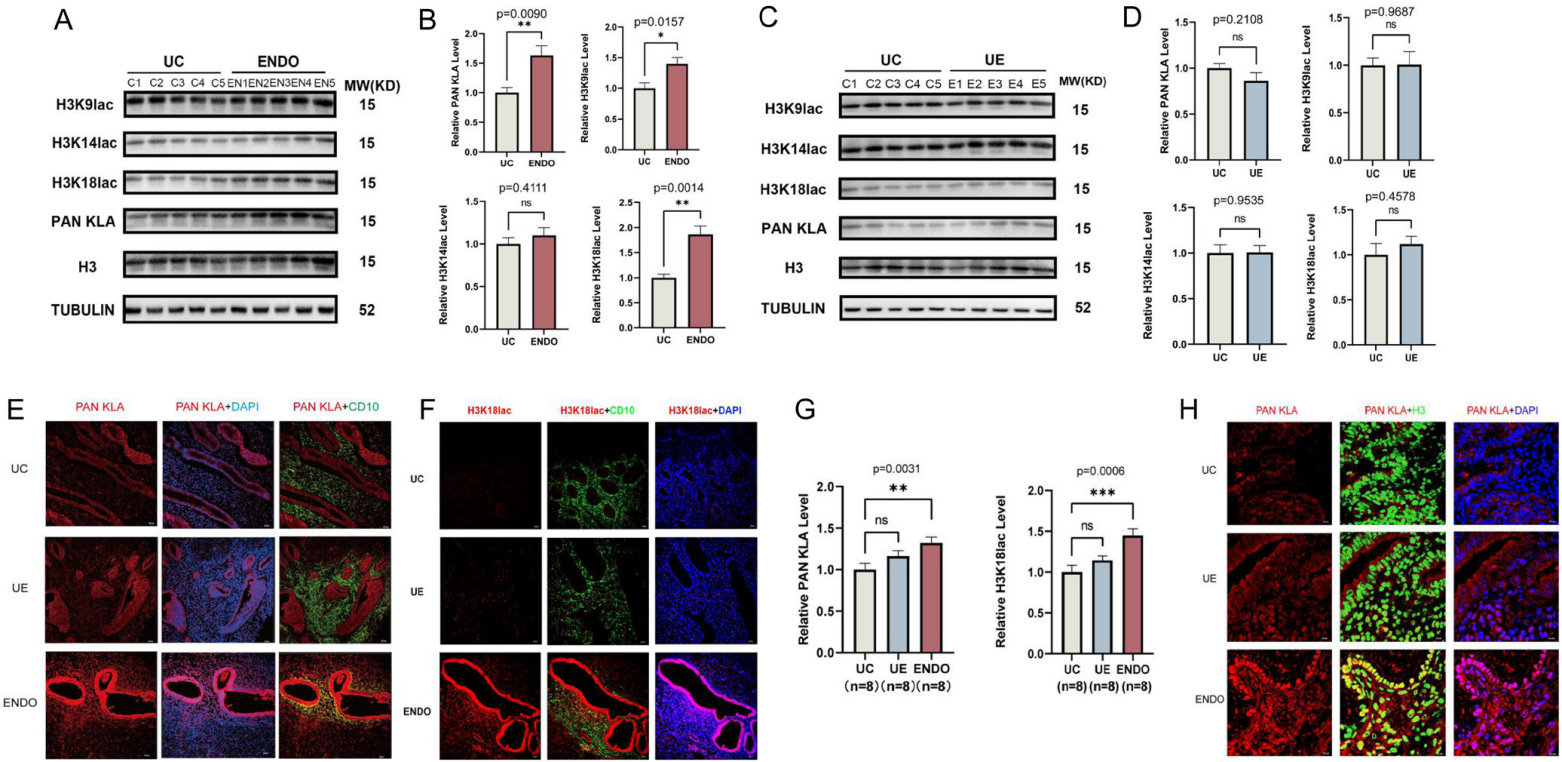
Elevated histone lactylation in ectopic endometrium of endometriosis. (A) Lactylation level was detected in UC and ENDO by western blot. Histone lactylation levels were significantly higher in the ENDO compared to the UC. (B) Quantitative analysis of (A). (C) Lactylation level was detected in UC and UE by western blot. There was no significant change in histone lactylation levels in the UE compared to the UC. (D) Quantitative analysis of (C). (E and F) Immunofluorescence was used to detect the degree of histone lactylation and H3K18lac in UC, UE, and ENDO. CD10 was used as a marker of endometrial stroma. There were no significant changes in histone lactylation levels and H3K18la expression in the UE compared to the UC. However, histone lactylation level and H3K18la were significantly elevated in the ENDO compared with the UC. Scale bar, 30 mm. (G) Statistical results of lactylation levels and H3K18lac levels in UC, UE, and ENDO. (H) Localization of lactyated proteins in UC, UE, and ENDO by using immunofluorescence. H3 was used as a marker of histone. Scale bar, 10 mm. The data are expressed as mean ± s.e.m. *P < 0.05, **P < 0.01, and ***P < 0.001; ns, no statistical difference.

Lactate, an essential metabolite produced by glycolysis, was recently shown to regulate the growth of cancer through lactate-mediated epigenetic histone modifications (lactylation) (Yu *et al.* 2021). Therefore, we examined histone lactylation in endometriosis by examining levels of histone lactylation in the UC, UE, and ENDO tissues. Quantifying protein levels of H3K9lac, H3K14lac, H3K18lac PAN lactylated lysine (KLA), and total histone 3 (H3) normalized to tubulin showed that lactylation levels in the UE did not differ from those in the UC (Fig. 2C and D). However, ENDO had higher histone lactylation levels relative to the UC (Fig. 2A and B). Compared to UC, the level of H3K18la was more greatly enriched in the ENDO relative to the UC compared with the other types of modifications (Fig. 2A and B) but did not differ between the UE and UC (Fig. 2C and D). Next, immunofluorescence staining was performed, revealing the ectopic endometrial tissue had greater levels of overall lactylated lysine and greater levels of histone H3K18lac (Fig. 2E and G). Co-staining of PAN KLA and H3 showed high co-localization in the nucleus of ENDO compared to the other groups (Fig. 2H), findings which are consistent with the greater levels of H3K18lac observed in ENDO compared with others. These findings revealed that increased histone lactylation was present in a significant fraction of cases of endometriosis and that this may have a role in the etiology of the condition.

### Long non-coding RNA H19 is associated with the presence of the Warburg effect in HESC

**Figure 3:**
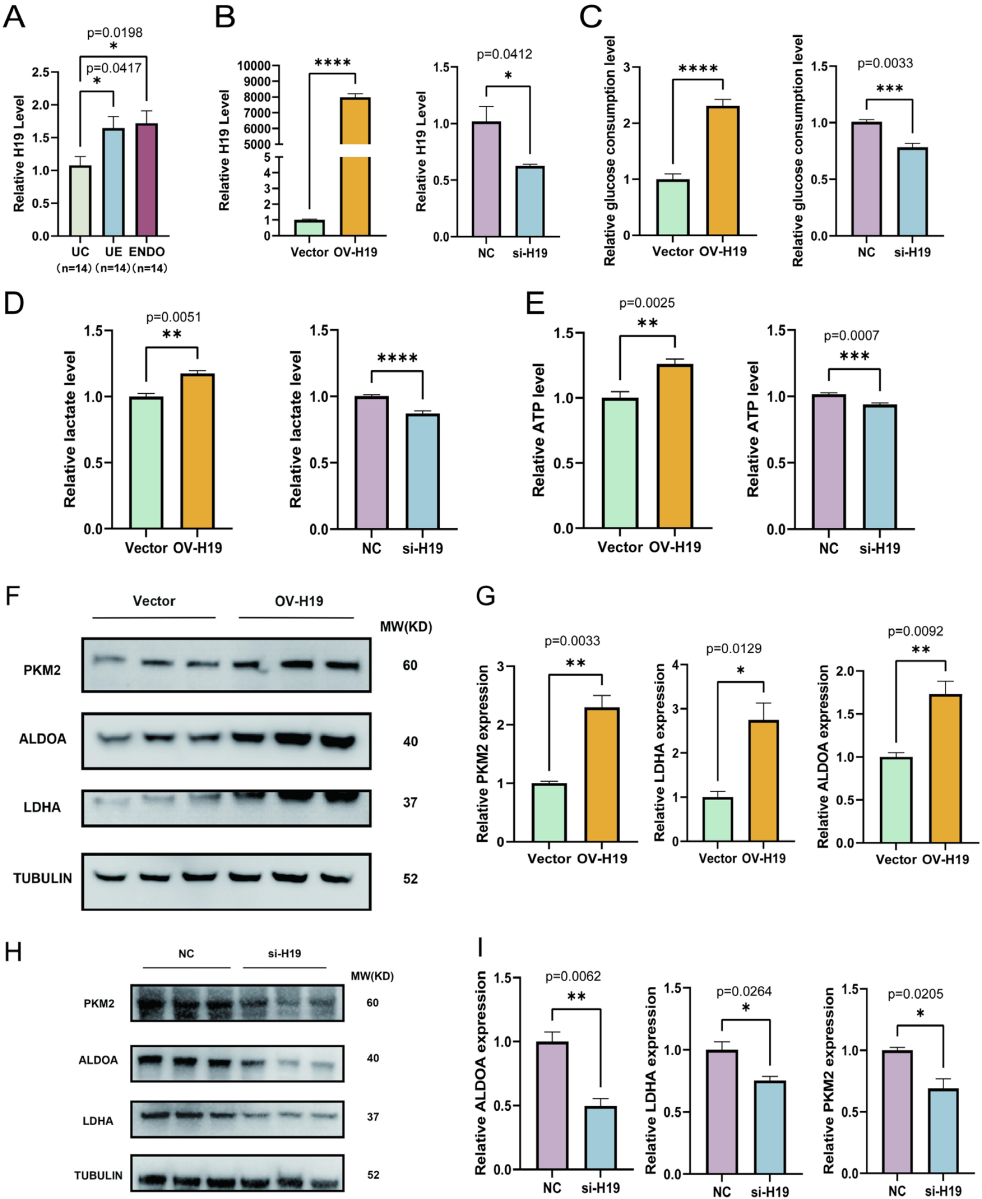
H19 was associated with aerobic glycolytic effects in HESCs. (A) Compared with UC, H19 expression is elevated in UE and ENDO. (B) H19 expression in the vector groups (Vector) and HESCs overexpressing H19 groups (OV-H19) after transfection of the H19 plasmid and in the control (NC) group and knockdown H19 (si-H19) group after transfection of the si-H19. (C and D) Glucose consumption level and lactate content in cell cultures after overexpression of H19 and knockdown of H19 were separately measured using the corresponding kits. (E) Detection of ATP production in indicated groups of cells. ATP production in HESCs was significantly higher after elevated H19 expression. (F) HESCs were transfected with overexpression H19 plasmid, and Vector and OV-H19 groups were examined for aerobic glycolysis-related protein expressions. The expression of aerobic glycolysis-related enzymes was significantly higher in HESCs after elevated H19 expression. (G) The protein level in Vector and OV-H19 groups was quantified and normalized to α-tubulin. The figure shows the expression of the OV-H19 group relative to the Vector group. (H,I) Using western blot to detect glycolysis-related proteins in NC and si-H19 groups. Decreased expression of H19 led to decreased expression of aerobic glycolysis-related enzymes. The figure shows the expression of the si-H19 group relative to the NC group. The data are expressed as mean ± s.e.m. *P < 0.05, **P < 0.01, ***P < 0.001, and ****P < 0.0001; ns, no statistical difference.

RT-qPCR was used to quantify the expression of H19 in normal endometrium, eutopic endometrium, and ectopic tissue. Compared to UC, levels of H19 were elevated in both the UE and ENDO (Fig. 3A). Due to challenges in acquiring primary eutopic endometrial stromal cells and primary ectopic endometrial stromal cells, and considering the high variability and low viability of cells from different sources, subsequent experiments were conducted using HESCs. In order to make our experiments more rigorous, both H19 overexpression and H19 knockdown conditions were examined. To determine the relationship between H19 expression and glycolytic activity, HESCs were transfected with either H19-overexpression plasmid or siRNA targeting H19 (Fig. 3B). The knockdown and overexpression experiments revealed a positive correlation between H19 and glucose consumption, lactate production, and ATP production (Fig. 3C, D, and E). We next examined the levels of glycolysis-related proteins using western blot. Compared to the vector control, protein levels of LDHA, PKM2, and ALDOA were found to be increased in cells overexpressing H19 (Fig. 3F and G), while H19 knockdown reduced LDHA, PKM2, and ALDOA levels (Fig. 3H and I). These results indicate that H19 expression regulates the level of aerobic glycolysis in HESCs.

### Increased H19 expression results in greater histone lactylation in HESCs

**Figure 4:**
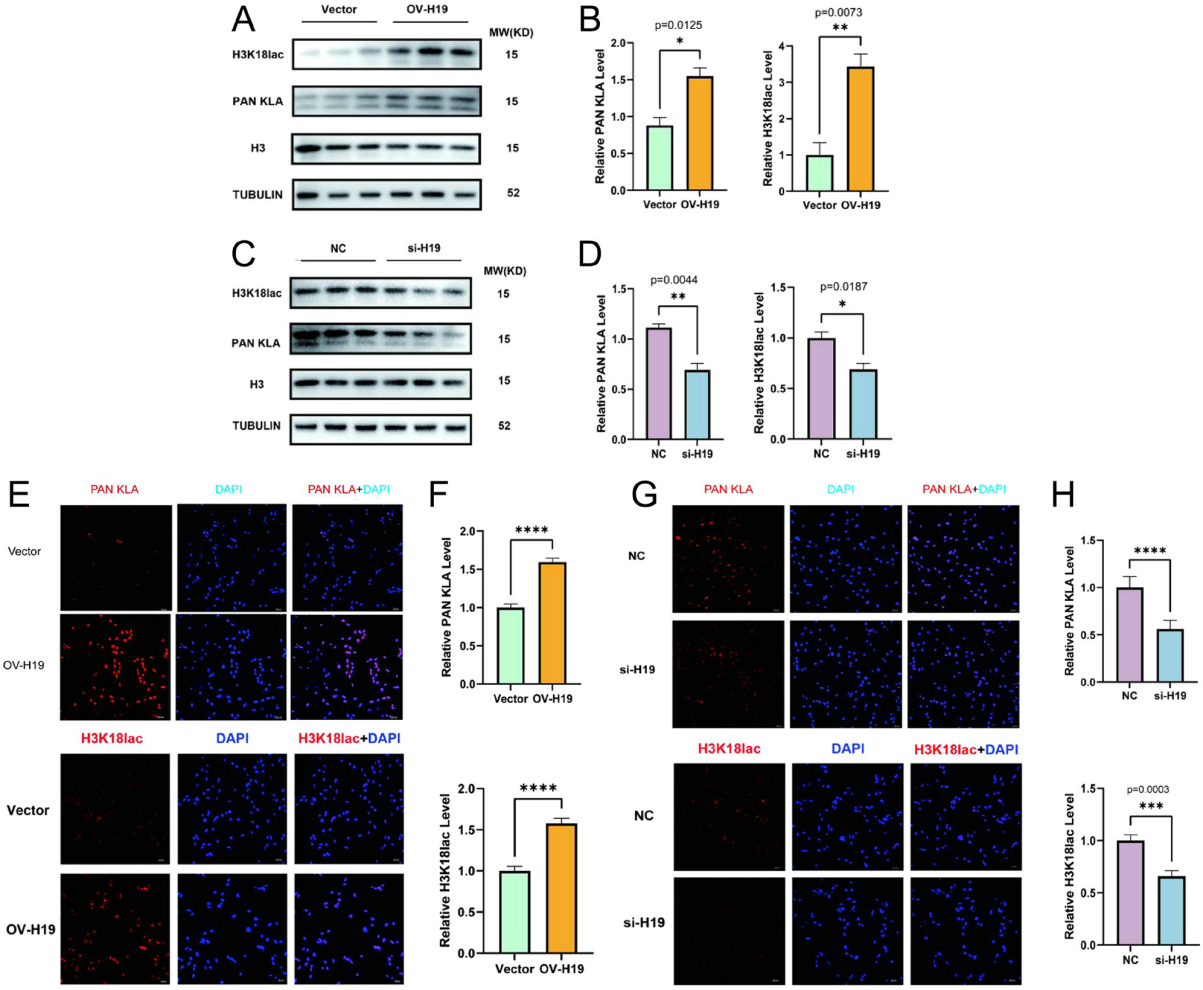
High expression of H19 led to increased histone lactylation in HESCs. (A) Western blot was used to detect the level of histone lactylation in the Vector and OV-H19 groups. Elevated expression of H19 increased the level of histone lactylation in HESCs. (B) Quantitative analysis of (A). (C and D) Densitometric analysis was performed to quantify and statistically compare lactylation levels in NC and si-H19 groups normalized to H3. Histone lactylation levels in HESCs decreased following a drop in H19 expression. (E) Histone lactylation and H3K18lac levels tested using immunofluorescence of Vector and OV-H19. Scale bar, 30 mm. (F) Quantitative analysis of (E). (G) Lactylation levels were visualized by immunofluorescence staining in NC and si-H19 groups. Scale bar, 30 mm. (H) Densitometric analysis was performed to quantify and statistically compare lactation levels in NC and si-H19. The data are expressed as mean ± s.e.m. *P < 0.05, **P < 0.01, and ***P < 0.001; ns, no statistical difference.

Using the knockdown and overexpression models, we examined histone lactylation levels after modulating H19 levels in HESCs. From western blotting, we observed histone lactylation levels to be positively correlated with H19 expression (Fig. 4A, B, C, and D). Immunofluorescence results showed that histone lactylation and H3K18lac levels were increased in HESCs overexpressing H19, along with an increased nuclear fluorescence signal (Fig. 4E, F, G, and H). These results demonstrated that elevating the expression of H19 results in increased HESC histone lactylation.

### Aerobic glycolysis and histone lactylation levels influence the proliferation and migration capacity of HESCs

**Figure 5:**
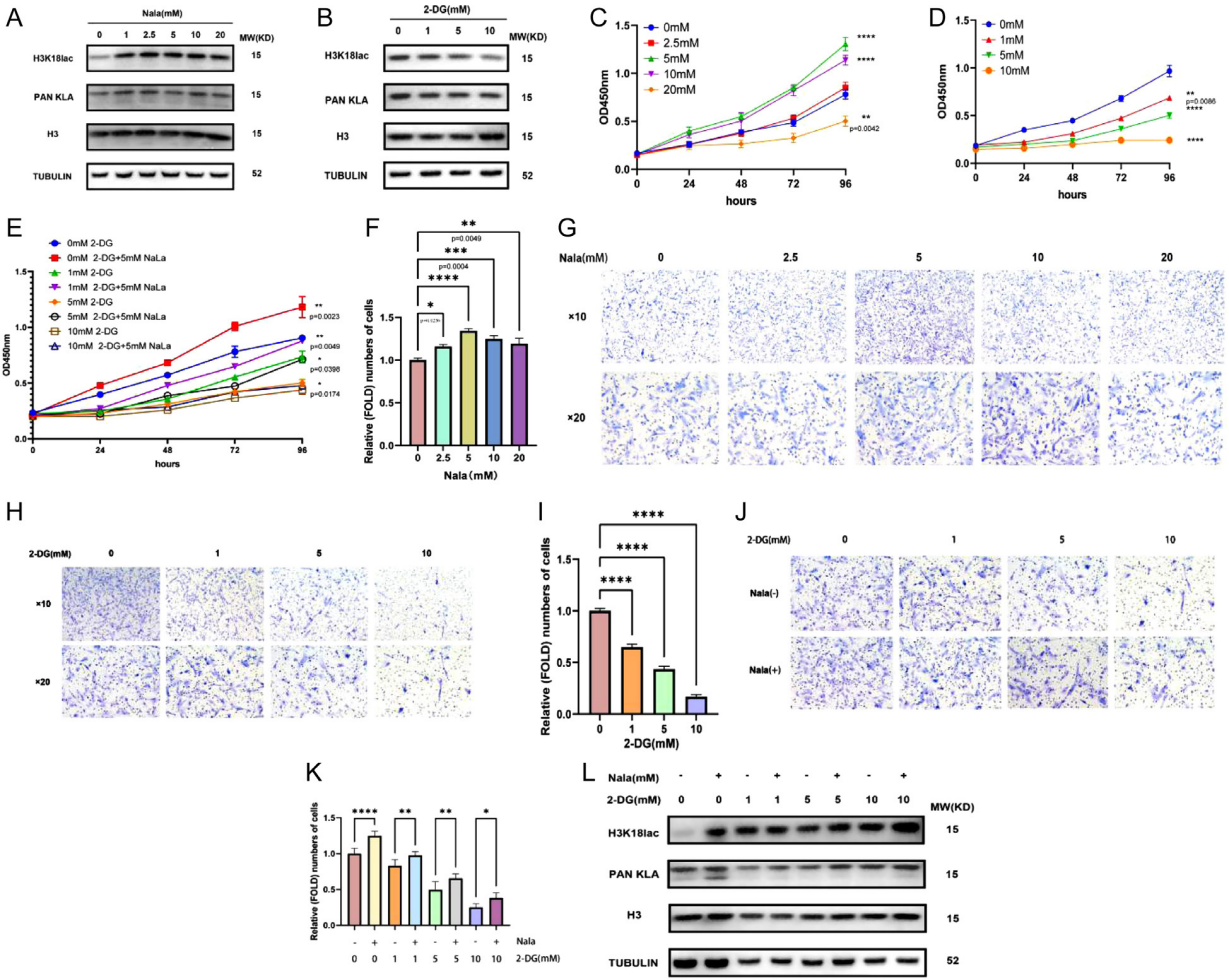
The Warburg effect and histone lactylation impacts on the proliferation and migration capacity of HESCs. (A and B) Lactylation and H3K18la levels were detected in HESCs cultured in different concentrations of NaLa and 2-DG by western blot. Histone lactylation levels were significantly elevated in HESCs in a high-lactic acid environment. (C and D) Using the CCK8 kit to detect cell proliferative capacity after the addition of NaLa and 2-DG. The proliferative capacity of HESCs was significantly increased in the high lactate environment. (E) Cell proliferative capacity after the addition of 2-DG and NaLa was detected with CCK8 kits. NaLa was able to partially restore the proliferative capacity of HESCs cells with the addition of 2-DG. (F–I) The migration ability of cells after the addition of NaLa and 2-DG was analyzed by transwell assay and statistical analysis. The migratory capacity of HESCs was significantly increased in the high-lactic acid environment. (J) The cell migration ability was analyzed by transwell assay after adding both NaLa and 2-DG. NaLa was able to partially restore the migratory ability of HESCs cells spiked with 2-DG. (K) Statistical analysis of cells in the transwell assay following the addition of both NaLa and 2-DG. (I) Histone lactylation levels in HESCs cells with both NaLa and 2-DG added were detected using western blot. NaLa was able to partially restore histone lactylation levels in HESCs cells incorporating 2-DG. The data are expressed as mean ± s.e.m.*P < 0.05, **P < 0.01, ***P < 0.001, and ****P < 0.0001; ns, no statistical difference.

Sodium lactate (NaLa) and 2-deoxy-d-glucose (2-DG) are widely recognized drugs that regulate aerobic glycolysis. However, the ability of NaLa and 2-DG to modulate histone lactylation levels had not been examined. We tested whether histone lactylation levels in HESCs were altered after treatment with NaLa) by western blot. The addition of different doses of NaLa led to an increase in histone lactylation in cells and increased lactylation of H3K18 (Fig. 5A). In contrast, the addition of 2-DG resulted in decreased histone lactylation (Fig. 5B). Therefore, NaLa was used to mimic the high-lactate environment observed in endometriosis, and 2-DG was used to reduce lactylation to determine the effect of glycolysis on endometriosis pathogenesis. Downstream of increased glycolysis and histone lactylation levels in HESCs treated with different concentrations of NaLa, we observed that the proliferative ability of treated cells was increased compared with cells lacking NaLa treatment, and 5 mM NaLa resulted in the greatest enhancement in cell proliferation (Fig. 5C). Transwell migration assays further showed that the migration ability of the cells was increased after the addition of NaLa, with the greatest levels of migration also observed after 5 mM NaLa treatment (Fig. 5F and G). After treating cells with different concentrations of 2-DG, compared to no 2-DG treatment, the proliferative (Fig. 5D) and migratory (Fig. 5H and I) capacity was decreased with increasing concentrations of 2-DG. In addition to cell proliferation, cell migration was inhibited by 2-DG, but the inhibition of cell proliferation and migration was partially rescued by co-treating cells with 2-DG and 5 mM NaLa (Fig. 5E, J, and K). Moreover, Co-treatment with NaLa and 2-DG raised levels of histone lactylation (Fig. 5L). Taken together, these findings suggest that the Warburg effect and elevated levels of histone lactylation increase the proliferative and migrative capacities of HESCs.

### Warburg effect and elevated histone lactylation existed in mice modeled with endometriosis

**Figure 6:**
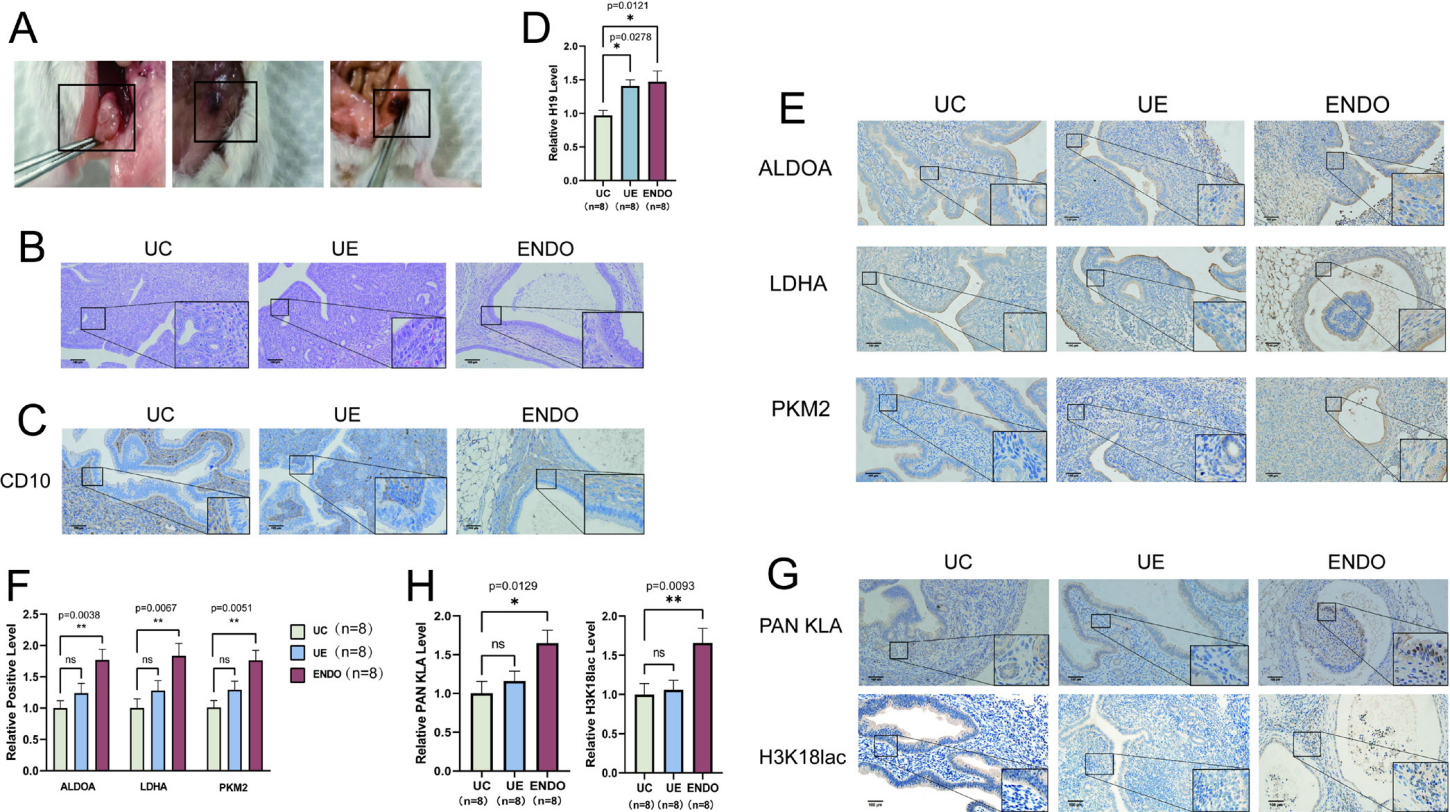
The Warburg effect and elevated histone lactylation in mice models of endometriosis. (A) The mice models of endometriosis were established using BALB/c female mice. (B) The HE staining of the control group mice uterus (UC), endometriosis mice with eutopic endometrium (UE), and ectopic endometrial tissue (ENDO). (C) Representative images of CD10 immunohistochemical staining in histopathologic sections of mice endometriotic tissues were shown. (D) Compared to UC, H19 expression was elevated in both UE and ENDO. (E,F) Immunohistochemical results and their quantitative analysis showed elevated expression of glycolytic key enzymes in the UE and ENDO groups compared to the UC group. (G and H) Histone lactylation and the level of H3K18lac were measured using immunohistochemistry in the tissues of UC, UE, and ENDO groups. Scale bar, 100 μm. The data are expressed as mean ± s.e.m.^*^P < 0.05, ^**^P < 0.01, ^***^P < 0.001, and ^****^P < 0.0001; ns, no statistical difference.

We constructed a mouse model of endometriosis (Fig. 6A) to examine glycolysis and histone lactylation *in vivo*. HE staining (17) and CD10 immunohistochemistry of endometrial tissues isolated from the model showed that the distribution of stroma and glandular tissue in lesions obtained from the model was similar to normal endometrial tissues, suggesting the utility of the mouse model (Fig. 6B and C). Next, we verified the expression of H19 in normal mouse endometrium (UC) and the eutopic endometrium (UE) and ectopic tissue (ENDO) obtained from the endometriosis model using RT-qPCR (Fig. 6D). H19 levels were elevated in both UE and ENDO in the endometriosis model compared to UC. Immunohistochemical staining demonstrated increased expression of glycolysis-related proteins in ENDO compared to the UC and UE (Fig. 6E and F). Meanwhile, it was observed that the level of histone lactylation was higher in ENDO tissue compared to UC and UE (Fig. 6G and H). These findings further demonstrated increased levels of histone lactylation in endometriosis. These findings provide more evidence that endometriosis is associated with increased levels of glycolysis and histone lactylation.

## Discussion

The pathological mechanism underpinning endometriosis remains unclear, and treatment is limited to hormonal therapy and surgery, which cannot wholly cure endometriosis (Zondervan *et al.* 2020). In a manner similar to tumor cells, ectopic endometrial stromal cells exhibit the Warburg effect, characterized by increased lactate generation and glucose consumption. Targeting glycolysis has therefore been one of the major focuses of recent studies examining endometriosis (Sun *et al.* 2022). PKM2, a rate-limiting enzyme of glycolysis, is preferentially expressed in proliferating cells, including cancer cells (Wang *et al.* 2021*a*). Ectopic endometrial stromal cells were found to have greater expression of PKM2 mRNA and greater PKM2 protein levels compared to normal endometrial stromal cells (Yao *et al.* 2021). LDHA, another rate-limiting enzyme for glycolysis, contributes to immune escape through the regulation of lactate production, promoting cell proliferation, and is highly expressed in various types of cancer (Feng *et al.* 2018). LDHA has also been implicated in the progression and prognosis of endometriosis (Zheng *et al.* 2021). ALDOA is a crucial mediator of glycolysis, gluconeogenesis, and the pentose phosphate pathway (PPP) (Fan *et al.* 2017). In colon cancer, ALDOA was shown to promote cell proliferation, clonogenicity, glycolysis, and PPP activity (Lin *et al.* 2022). In contrast to the normal endometrial and eutopic endometrium, our investigation demonstrated increased expression of ALDOA, PKM2, and LDHA in ectopic endometriosis. These findings suggest that increased expression of glycolytic enzymes underlies the increased glycolysis observed in endometriosis. However, aerobic glycolysis levels were not increased in eutopic endometrium, and we hypothesized that considering the patient populations under research are highly heterogeneous, there is frequently a substantial level of inter-study variability among endometriosis-associated parameters.

Histones contain multiple types of posttranslational modifications, including acetylation, methylation, phosphorylation, ubiquitination, and SUMOylation, which have been well described (Pan *et al.* 2022). Recently, advances in high-sensitivity mass spectrometry have revealed a variety of additional histone acylation marks derived from cellular metabolites, including propionylation, butyrylation, succinylation, and malonylation (Lv *et al.* 2023). Recently, Zhang *et al.* identified lactate-derived histone lactylation as an epigenetic modification that enhances gene transcription (Zhang *et al.* 2019). As a novel histone modification, histone lactylation was subsequently found to be essential for tumorigenesis (Jiang *et al.* 2021). However, its role in endometriosis has not been reported. In this study, we describe increased levels of aerobic glycolysis in the ectopic endometrium of endometriosis, resulting in increased production of lactic acid that can serve as a substrate for histone lactylation (Chen *et al.* 2021). These observations prompted our examination of a potential role for histone lactylation in the development of endometriosis. In this study, we show increased histone lactylation, specifically H3K18lac, in the ectopic endometrium of endometriosis patients. In conclusion, we show that histone lactylation is elevated in endometriosis. The role and mechanism of histone lactylation are complex, and more extensive and in-depth studies for its role in endometriosis are needed.

Long non-coding RNAs promote the reprogramming of gluconeogenesis in tumors, mainly through direct regulation of metabolism-related enzymes and indirect targeting of oncogenes (Deng *et al.* 2021). H19 has been shown to promote aerobic glycolysis, cell proliferation, and immune escape in gastric cancer cells by acting through the miR-519d-3p/LDHA axis (Sun *et al.* 2021). Our previous study showed that lncRNA H19 expression was elevated in the eutopic endometrium of patients with endometriosis (Xu *et al.* 2019). Meanwhile, increased lncRNA H19 expression has been reported in the ectopic endometrium of patients with endometriosis (Liu *et al.* 2020). These observations prompted our hypothesis that enhanced aerobic glycolysis in the ectopic endometrium of endometriosis might occur downstream of elevated H19 expression. In this study, we determined that manipulating H19 levels in HESCs coul alter aerobic glycolysis levels, with increased H19 expression leading to increased levels of aerobic glycolysis in ectopic endometrial stromal cells. Elevated H19 expression resulted in increased expression of enzymes related to aerobic glycolysis enzymes as well as increased glucose uptake, lactate production, and ATP synthesis. These findings suggest that elevated levels of aerobic glycolysis in the ectopic endometrium of endometriosis are directly caused by increased H19 expression. Currently, the mechanistic underpinnings for how H19 regulates downstream molecules to control glucose metabolism in endometriosis stromal cells is unclear.

Since both H19 expression and aerobic glycolysis levels are elevated in the ectopic endometrium of endometriosis patients, and lactate produced by aerobic glycolysis functions as a key substrate in the lactylation of histones, we hypothesized that high H19 expression could promote elevated histone lactylation levels in endometriosis. Cellular immunofluorescence and western blotting revealed a positive correlation between H19 expression and histone lactylation levels, showing that high expression of H19 elevates the levels of aerobic glycolysis leading to increased histone lactylation, and that H19 might be a critical regulatory component linking aerobic glycolysis and histone lactylation in endometriosis. Currently, the only growth data available were produced using an *in vitro* stromal cell model, which does not accurately recapitulate to accurately capture the complexities of the cellular interactions observed *in vivo*.

To further explore the effects of aerobic glycolysis and histone lactylation levels on the development of endometriosis, we examined the changes in the proliferation and migration ability of HESCs after modulating histone lactylation levels by treating cells with either NaLa or 2-DG. These experiments revealed that the proliferation and migratory capacity of HESCs were significantly enhanced upon elevation of aerobic glycolysis and histone lactylation. Similarly, in colon cancer, LPS was shown to promote histone lactoylation on the LINC00152 promoter, leading to increased expression and promoting the invasion and migration of colon cancer cells (Wang *et al.* 2022). This is strong evidence that aerobic glycolysis and histone lactylation are important regulatory links in the disease progression of endometriosis.

To further explore this mechanism *in vivo*, we created a mouse model of endometriosis. Similar mouse models have previously been described (Lai *et al.* 2021). H19 levels were found to be increased in both the eutopic endometrium and ectopic endometrium of the endometriosis model, observations that are consistent with our previous findings in human tissues. When compared to the normal mouse endometrium and the eutopic endometrium of endometriosis model mice, immunohistochemical data showed higher levels of glycolysis-related enzymes and histone lactylation in the ectopic endometrium tissue. These observations support our previous experimental findings and demonstrate H19 overexpression raises glycolysis and, in turn, increases histone lactylation in endometriosis.

## Conclusion

Taken together, the results of our study demonstrate that high expression of H19 in endometriosis promotes enhanced levels of aerobic glycolysis and histone lactylation. Our study highlights the importance of glucose metabolism in endometriosis and demonstrates for the first time that elevated levels of histone lactylation occur in the ectopic endometrium of endometriosis. Our findings suggest abnormal glucose metabolism and increased histone lactylation in endometriosis promote disease pathogenesis, providing a novel perspective on disease etiology and therapeutic approaches to endometriosis. Nevertheless, the mechanism by which H19 increases levels of aerobic glycolysis and histone lactylation in endometriosis remains unknown and should be elucidated by future investigations.

## Supplementary Materials

Supplementary Tables

## Declaration of interest

The authors declare that there is no conflict of interest that could be perceived as prejudicing the impartiality of the study reported.

## Funding

This study was funded by the National Natural Science Foundation of Chinahttp://dx.doi.org/10.13039/501100001809 (grant no. 82071617), the Natural Science Foundation of Shandong Provincehttp://dx.doi.org/10.13039/501100007129 (grant no. ZR2020QH058), and Medical and Health Science and Technology Development Project of Shandong Provincehttp://dx.doi.org/10.13039/501100019446 (grant no. 202105010952).

## Ethics approval and patient consent

All animal procedures were approved by the Reproductive Hospital Affiliated to Shandong University Institutional Review Board ((2020) IRB No. (14)). The experiments were performed complying with the Declaration of Helsinki, following the experimental and welfare guidelines of Shandong University. Written informed consent was obtained from all participants during the presentation for hysteroscopy treatment.

## Data availability

All data are available in the main text. They are also available on reasonable request to corresponding authors.

## Author contribution statement

XW designed the study. JZ and ML performed the experiments. ZX and YD were involved in data analyses and interpretation. ZX and XD revised the work critically for important intellectual content. The first draft of the manuscript was written by XW and LY. All authors commented on previous versions of the manuscript. All authors read and approved the final version of the manuscript.

## Acknowledgements

We thank our patients and all participants of the study. Thanks are due to Professor Yanbo Du from Shandong University for guiding our experimental design. Thank are also due to our colleagues at Medical Integration and Practice Center, Shandong University, Jinan, Shandong, China, for assistance with sample collection. We are grateful to Dr Lei Yan and other colleagues for their help with the experimental protocol.
