# Supplementary material for: Highly expressed lncRNA H19 in endometriosis promotes aerobic glycolysis and histone lactylation
Source: Reproduction. 2024 Jul 2;168(2):e240018. doi: 10.1530/REP-24-0018 (PMC11301422; doi:10.1530/REP-24-0018)
Supplement: Supplementary Tables [file supplementary_tables.pdf]

Supplemental Table S1

Characteristics of patients

	Control group	Endometriosis group	*P* value
Age（yrs）	33.25±2.7726	33.45±3.2011	0.5299
BMI（kg/m^2）	21.71±1.2250	20.62±1.7234	0.3325
FSH(IU/L)	6.10±1.9651	7.14±1.4543	0.3386
LH(IU/L)	3.94±1.2563	5.03±1.9816	0.3462
PRL(ng/mL)	15.17±3.1238	14.87±3.654	0.4146
E2(pg/ml)	34.58±3.9723	48.45±7.0569	0.0184*

Characteristics of patients in WB

	Age (yrs)	BMI (kg/m^2)	FSH (IU/L)	LH (IU/L)	PRL (ng/mL)	E2 (pg/ml)
C1	36	21.38	4.73	5.07	13.7	24.1
C2	34	20.26	6.41	5.23	17.4	14.8
C3	35	19.33	9.77	4.28	14.56	23.6
C4	29	22.11	5.88	2.59	13.2	40.3
C5	33	21.47	6.47	3.91	15.66	24.6
C6	35	21.89	6.04	2.89	15.5	46.4
E1	32	18.21	7.08	1.84	12.5	47.2
E2	36	20.83	4.64	5.93	18.11	42.5
E3	33	20.04	7.31	3.06	16.7	58.8
E4	35	21.15	6.71	2.96	13.15	37.7
E5	35	22.03	8.97	2.91	12.2	32.6
E6	34	21.76	5.82	4.19	13.92	38.8

Supplemental Table S2

The sequences of primers

Primer	Sequence
H19(Homo) Forward	ACGTGACAAGCAGGACATGA
H19(Homo) Reverse	TAAGGTGTTCAGGAAGGCCG
H19(Mus) Forward	CAGAGCAAAGGCATCGCAAA
H19(Mus) Reverse	GCTCCCCTTTATCCGACCAG
ACTB(Homo) Forward	GGCACCACACCTTCTACAATGAGC
ACTB(Homo) Reverse	GATAGCACAGCCTGGATAGCAACG
ACTB(Mus) Forward	GTGACGTTGACATCCGTAAAGA
ACTB(Mus) Reverse	GCCGGACTCATCGTACTCC
